# The *Caenorhabditis elegans* Homeobox Gene *ceh-19* Is Required for MC Motorneuron Function

**DOI:** 10.1002/dvg.22365

**Published:** 2013-01-12

**Authors:** Huiyun Feng, Ian A Hope

**Affiliations:** School of Biology, Faculty of Biological Sciences, The University of LeedsLeeds, LS2 9JT, United Kingdom

**Keywords:** Caenorhabditis elegans, transcription factors, homeodomain, nerve cells, pharynx

## Abstract

Simplicity has made *C. elegans* pharyngeal development a particularly well-studied subject. Nevertheless, here we add the previously uncharacterized homeobox gene *F20D12.6/ceh-19* to the set of transcription factor genes involved. GFP reporter assays revealed that *ceh-19* is expressed in three pairs of neurons, the pharyngeal pace-maker neurons MC, the amphid neurons ADF and the phasmid neurons PHA. *ceh-19(tm452)* mutants are viable and fertile, but grow slightly slower, produce less progeny over a prolonged period, and live longer than the wild type. These phenotypes are likely due to the moderately reduced pharyngeal pumping speed arising from the impairment of MC activity. MC neurons are still born in the *ceh-19* mutants but display various morphological defects. *ceh-19* expression in MC is completely lost in progeny from animals subject to RNAi for *pha-4,* which encodes an organ-specifying forkhead transcription factor. CEH-19 is required for the activation in MCs of the excitatory FMRFamide-like neuropeptide-encoding gene *flp-2*. A regulatory pathway from *pha-4* through *ceh-19* to *flp-2* is thereby defined. The resilience of MC identity in the absence of CEH-19 may reflect the buffering qualities of transcription factor regulatory networks. genesis 51:163–178, 2013. © 2013 Wiley Periodicals, Inc.

## INTRODUCTION

The *C. elegans* pharynx is an anatomically self-contained structure dedicated to ingestion and transportation of bacteria into the intestine (Albertson and Thomson, 1976; Avery and Horvitz, 1989). The entire structure is surrounded by and isolated from the rest of the worm by a basement membrane (Sulston *et al*., 1983). The feeding behavior is accomplished by two distinct types of muscle contractions, namely pumping and peristalsis. Pumping ingests and concentrates bacteria in the anterior lumen and is followed by peristaltic contractions, which bring ingested bacteria through the isthmus (Avery and Shtonda, 2003). Feeding is regulated and modulated by the 20 pharyngeal neurons, almost completely independently of neuro-muscular activity elsewhere in the animal (Avery and Horvitz, 1989). Extensive laser ablation experiments have shown that the nervous system is not essential for pumping, but is important for normal feeding, growth rate, and fertility (Avery and Horvitz, 1989). Pharyngeal muscles pump even without the stimulation from neurons, but the pace of pumping is controlled by the motor neurons MC and M3, which initiate and terminate the muscle action potentials respectively (Raizen *et al*., 1995). The MC neuron is an excitatory cholinergic neuron. Its firing triggers a pharyngeal muscle action potential via the release of acetylcholine, which acts on a muscle nicotinic receptor. It is said to be necessary and probably sufficient for rapid pharyngeal pumping (Mukhopadhyay *et al*., 2007; Raizen *et al*., 1995). A third motor neuron, M4, is responsible for peristalsis of the isthmus; its inactivation has little effect on pumping but eliminates peristalsis causing animals to arrest with a “stuffed pharynx” (Avery and Horvitz, 1989).

Homeobox genes have been shown to be required for pharynx morphogenesis and cell differentiation. Notably, several NK-2 class homeodomain factors, including PHA-2, CEH-2, CEH-22, and CEH-24, are each required, independently of or in combination with other transcription factors, for target gene expression in and specification of one or a few of the 8 sets of pharyngeal muscles (pm1-pm8) (Harfe and Fire, 1998; Mango, 2009; Morck *et al*., 2004; Okkema and Fire, 1994; Okkema *et al*., 1997). NK-2 class homeodomain factors also function in the pharyngeal nervous system. For example, *ceh-2* is expressed in the NSM and M3 neurons, and in a *ceh-2* deletion mutant M3 was generated but its activity was substantially reduced, indicating *ceh-2* acts in a late differentiation step for M3 development (Aspock *et al*., 2003). Similarly, CEH-28 is required for M4 function; *ceh-28* inactivation results in irregularly spaced and sized M4 synapses in the isthmus, and frequent and prolonged peristalses (Ray *et al*., 2008).

**FIG. 1:**
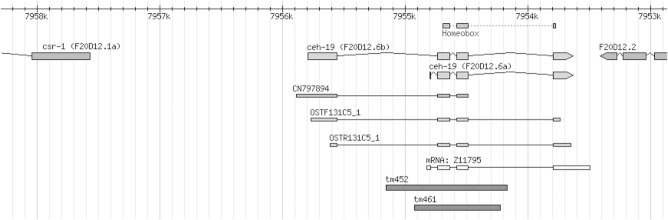
The *ceh-19* gene model. In WormBase, *ceh-19* is annotated to encode two transcripts, ceh-19a (F20D12.6a) and ceh-19b (F20D12.6b), based on the cDNA clones Z11795 and CN797894, and ORFeome sequence tags (OSTs). The extent of the homeobox and both deletion alleles, *tm452* and *tm461*, is indicated. *ceh-19* is located between the genes *csr-1 (F20D12.1)* and *F20D12.2*, both transcribed in the opposite direction to *ceh-19*. The scale is in kb.

Although MC has long been known to be the pacemaker neuron, regulatory factors critical for its specification and action, other than the FoxA transcription factor PHA-4 which is required broadly for pharyngeal cell fate specification, had not been identified. Here we report that a previously uncharacterized homeobox gene, *F20D12.6/ceh-19* (Fig. 1), is specifically expressed in MCs from late embryogenesis through to adulthood. Well-fed, healthy animals bearing the *ceh-19(tm452)* or *ceh-19(tm461)* deletion mutations, that both remove most of the homeobox, displayed a moderate reduction of pharyngeal pumping speed. In *ceh-19(tm452)* mutants, the MC cells are generated, but with obvious axonal morphological defects, suggesting CEH-19 is required for proper specification of the MC neuronal type. Reporter fusions for *ceh-19* also directed expression in two pairs of sensory neurons, the amphid and phasmid neurons ADF and PHA. We found that the *ceh-19* expression level in ADF was up-regulated in wild type dauer animals, indicating a potential link of this homeoprotein to the *C. elegans* dauer and/or aging pathways.

## RESULTS

### *F20D12.6/ceh-19* Expression Pattern

In (WormBase) (http://www.wormbase.org), *ceh-19* is annotated to encode two transcripts, ceh-19a and ceh-19b. The two transcripts would produce proteins of 122 and 199 amino acids, respectively, with exons 2, 3, and 4, encoding the carboxyl terminal 119 amino acids including the homeodomain, in common, but each with unique first exons (Fig. 1). The *C. elegans* Promoterome project (Reece-Hoyes *et al*., 2007) had generated a *ceh-19b^prom^::gfp* fusion containing the 1.5 kb region upstream of the *ceh-19 b* start codon. In strains UL2701, carrying this reporter gene fusion as an extrachromosomal array, and UL2702 and UL2703, containing chromosomally integrated arrays of the same fusion, this assayed promoter region had been described as driving GFP expression in four neurons in the head and two neurons in the tail region, from 3-fold embryos until the adult stage.

**FIG. 2:**
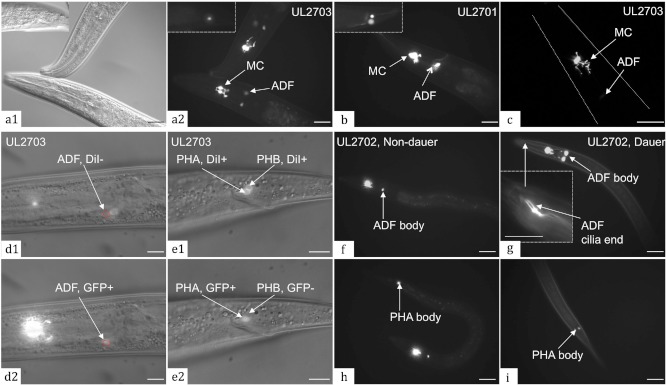
Expression of the *ceh-19b^prom^::gfp* fusion. GFP distributions were observed in UL2701 (**b**), UL2702 (**f, g, h, i**) and UL2703 (**a, c, d, e**). Three pairs of neurons, two pairs in the head, MC and ADF, (**a, b**) and one pair in the tail region, PHA, (**a, b** insets) expressed GFP. DIC optics (**a1**) reveals the position of fluorescence (**a2**) within the same animal. The path of axon projections was determined using confocal Z-sections, as in this projection (**c**). The amphid neurons ADF (circled in red), expressing the GFP (**d2**), do not take up DiI (**d1**). In the tail, DiI stained both phasmids PHA and PHB (**e1**), whereas only PHA expressed GFP (**e2**). The red fluorescence (**d1, e1**) and green fluorescence (**d2, e2**) are superimposed upon the DIC image. The ADF signal increased in the dauer (**g**) compared to the L3 (**f**), both images being captured at the same magnification and with a 1 second exposure. The dual ciliated end of ADF is clearly visible in dauers at high magnification (**g** inset). The GFP signal in PHA is similar in L3s (**h**) and dauers (**i**). Scale bars represent 25 µm in **a1/2, b, f, g, h, i**, 10 µm in **d1/2, e1/2, g** inset, and 16 µm in **c**.

Closer inspection of UL2701, UL2702, and UL2703 revealed that the cell bodies of two of the GFP expressing neurons in the head were located in the anterior bulb of the pharynx. There are only 20 neurons within the pharynx and from the axonal morphology these neurons were identified unambiguously as MCL/R (Fig. 2a–c).

The other two GFP expressing cells in the head had cell bodies outside of the pharynx, just anterior to the posterior pharyngeal bulb, with dendrites extending to the head tip and therefore were identified as amphids. Specific identity was narrowed down by their dual ciliated sensory endings (Fig. 2g inset), a morphological feature of only ADFL/R and ADLL/R. Furthermore as the GFP expressing cells did not take up externally delivered DiI (Fig. 2d1), which stains ASI, ADL, ASK, AWB, ASH, and ASJ, the expressing cells were identified as ADFL/R. In addition, the GFP expression increased in this pair of amphids in the dauer stage (Fig. 2g) and ADFs control entry into the dauer stage (Bargmann and Horvitz, 1991b), suggesting a connection between *ceh-19* regulation and dauer formation. The GFP signal was much higher in MCs than in ADFs during normal developmental stages (Fig. 2a, c, d2, f) whereas the strength of signal in the two neuron types was approximately the same in the dauer stage due to the considerable increase of signal within ADFs (Fig. 2g). In UL2701, which contains the transgene as an extrachromosomal array, the difference in intensity in non-dauer stages was not as apparent (Fig. 2b).

The two cells in the tail showing GFP expression have cell bodies located just behind the rectum and short processes in both anterior and posterior directions (Fig. 2b inset, h, i), a morphology suggestive of the phasmids neurons, PHAL/R or PHBL/R. DiI filling, which stains both of these phasmid neuron types revealing their relative positions, confirmed that the pair of neurons expressing GFP in the tail is the more anterior pair, PHAL/R (Fig. 2, E1/2). The extrachromosomal array in UL2701 directed stronger GFP expression in PHA than the integrated array in UL2702 and UL2703. In all three strains there was no apparent difference of signal in PHA between non-dauer and dauer animals (Fig. 2h, i). Although the tail neural anatomy is substantially modified in the male, no male specific expression was observed for the *ceh-19b^prom^::gfp* fusion in any of the three transgenic strains.

**FIG. 3:**
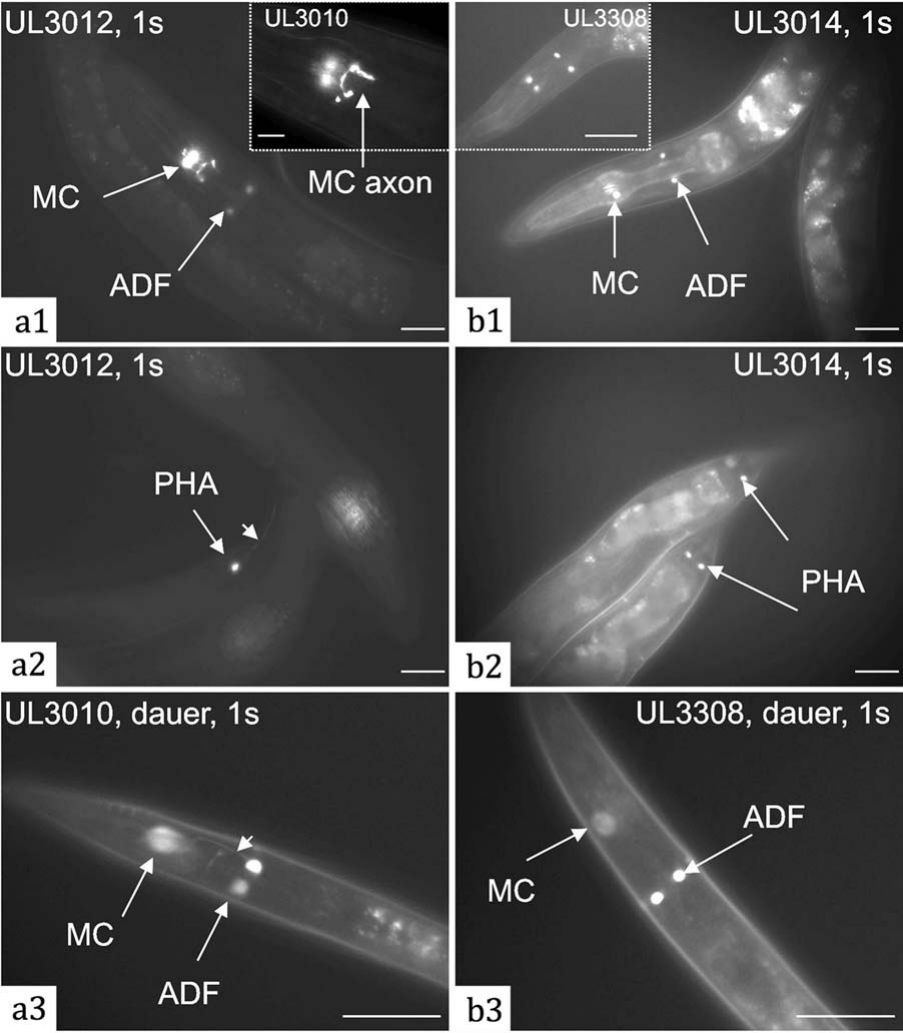
GFP expression of fosmid-based reporter fusions for *ceh-19b*. The recombineered fosmid fUL#HF005.1, with the *ceh-19b* protein coding region replaced by *gfp*, drove GFP expression in MCL/R, ADFL/R and PHAL/R in UL3010 and UL3012 (**a1–3**). Expression was detectable in axons of MC and PHA (**a1** inset, **a2** arrow head). The recombineered fosmid fUL#HF004.1, with *gfp* inserted immediately before the stop codon of *ceh-19b*, also drove GFP expression in MCL/R, ADFL/R and PHAL/R, in UL3014 and UL3308 (**b1–3**), but to a low level and with nuclear-localization. Expression in ADFs increased in dauer animals such that the axon of ADF was visible in UL3010 (**a3**, arrow head) and ADF nuclei became much brighter in UL3308 (**b3**). Bars represent 25 µm in **a1–3, b1–3** and inset, and 10 µm in **a1** inset.

We further generated two *gfp* fusions for *ceh-19b* in a large genomic background by recombineering the fosmid WRM0620bD04. This fosmid contains 18,586 bp upstream and 15,366 bp downstream of *ceh-19* and would be expected to contain all *ceh-19* regulatory elements. Strains UL3010, UL3011, and UL3012 independently transformed with the fosmid fUL#HF005.1, with the *ceh-19b* protein-coding region in WRM0620bD04 replaced precisely by *gfp*, showed consistent GFP expression in ADFL/R, MCL/R, and PHAL/R (Fig. 3a1/2), as obtained with the promoter reporter fusion. GFP was distributed throughout the cell body and along the neuronal axon as expected for a transcriptional fusion, but the signal was lower than with the plasmid-based fusion. The recombineered C-terminal translational fusion, with *gfp* inserted immediately before the *ceh-19* termination codon (fUL#HF004.1) to tag both *ceh-19a* and *ceh-19b*, drove GFP expression in apparently the same pairs of neurons, i.e. ADFL/R, MCL/R, and PHAL/R, in 2 independent strains (UL3014, UL3308) (Fig. 3b1/2). GFP was localized to the cell nucleus only, as expected for a transcription factor fusion protein, and at very low level, making cell identification less certain. For both recombineered reporter fusions, expression in ADFL/R was very low through the normal life cycle but increased greatly in dauer animals (Fig. 3a3, b3), as for the plasmid-based fusions, allowing the cell processes to be seen more clearly (Fig. 3a3). This increase was not observed in animals that had simply been starved (data not shown). DiI filling on these strains was consistent with ADF and PHA identification.

A reporter fusion was constructed to assay the expression pattern of the annotated ceh-19a transcript, supported by the single EST Z11795 and SAGE tags for exon 1 (Naito *et al*., 1992). The *gfp* reporter was fused to the 2770 bp region upstream of the *ceh-19a* start codon, including the start of transcript b and the entire intergenic region, by Gateway recombinational cloning, creating pUL#HF053. However, in two strains transformed with this plasmid no GFP expression was observed, throughout the normal life cycle or in the dauer, suggesting the ceh-19a transcript may not be functional or may only be expressed in circumstances not assayed. The absence of additional expressing cells for the C-terminal translational fusion is also consistent with *ceh-19a* not adding to the expression arising from *ceh-19b*.

### Molecular Phylogeny of CEH-19 and Its Homologues

Within the *C. elegans* genome, *ceh-30*, *ceh-31*, *tab-1,* and *ceh-1* have the highest similarity to *ceh-19b* by BLAST. Each of the five paralogues has one orthologue (best reciprocal BLAST match) in *Caenorhabditis briggsae* and *Caenorhabditis remanei*, suggesting conserved functional importance*. CG13424* and *CG10604* (*bsh*, brain specific homeobox) are the *Drosophila melanogaster* genes most similar to *ceh-19b*. *CG13424* has not been functionally characterized. *bsh* is expressed in approximately 30 cells in each brain hemisphere of the *Drosophila* embryo but a *bsh* deletion showed no dramatic changes in embryonic brain morphology and therefore its function in the *Drosophila* brain is unknown (Jones and McGinnis, 1993). Two BarH-like homeobox genes, *BARHL1* and *BARHL2* are the closest homologues of *ceh-19b* in the human genome.

**FIG. 4:**
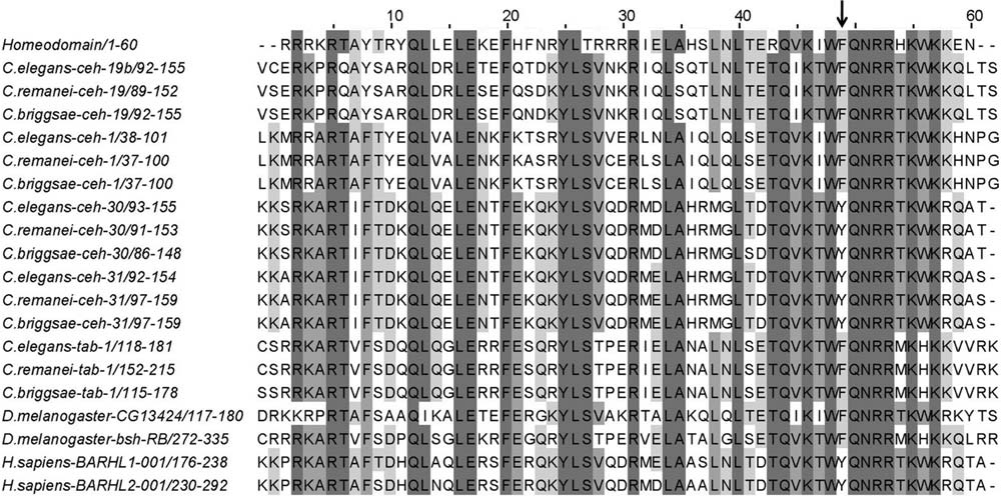
Protein sequence alignment of the homeodomains of *C. elegans* CEH-19 and its homologues from *C. briggsae*, *C. remanei*, *D. melanogaster*, and *H. sapiens*, in comparison with the more general consensus homeodomain. The arrow points to position 49 of the homeodomain, which is a characteristic tyrosine (Y) residue for the typical BarH homeoproteins rather than the more common phenylalanine (F). Alignment was performed using ClustalW software and edited with Jalview 2.5.1.

WormBase has CEH-19 placed in the BarH-like TF family, presumably based on its closest human homologues. However, BarH family members normally possess a highly conserved tyrosine (Y) residue at position 49 of the third helix of the homeodomain, as in the homeodomains of *Caenorhabditis* CEH-30 and CEH-31 and human BARHL1 and BARHL2 but not CEH-19 (Fig. 4) (Peden *et al*., 2007; Reig *et al*., 2007). In addition, typical BarH type homeoproteins have one or more FIL domains, enriched in phenylalanine (F), isoleucine (I), and leucine (L) residues, within the amino terminal region (Germán Reig *et al*., 2007) and a conserved 22-amino-acid BARC motif, immediately downstream of the homeodomain (Schwartz and Horvitz, 2007), which are also absent from CEH-19. Therefore CEH-19 does not appear to be a *C. elegans* orthologue of vertebrate BarH-like proteins. With no function yet determined for the potential CEH-19 orthologues in *Drosophila* either, no clues as to CEH-19 function can be inferred from other species.

Instead of the FIL domain, the N-termini of CEH-19 across the *Caenorhabditid* species are enriched for the acidic residues aspartate (D) and glutamate (E) typical of a transcription activation domain (Willmore, 2004). Acidic activation domains from many transcription factors have been shown to interact with the general transcription factors TFIIB, TFIID, and TFIIH of the basal transcription machinery (Xiao *et al*., 1994). Lack of this N-terminal region in CEH-19a makes it less likely that this short isoform, if expressed, could function as a transcription activator, but it may still make sequence specific DNA interactions and form homodimeric complexes, or heterodimeric complexes with other factors, using its homeodomain and carboxy-terminal region.

### ceh-19 (tm452) Has Reduced Pharyngeal Pumping Speed

To investigate the function of *ceh-19*, two *C. elegans ceh-19* mutant alleles were obtained and examined for a phenotype altered from the wild type. The *ceh-19(tm452)* and *ceh-19(tm461)* alleles, generated by TMP/UV mutagenesis, have 993 bp and 701 bp deletions, respectively. Both alleles lack the two middle exons of *ceh-19b* and the first three exons of *ceh-19a* (Fig. 1). This was verified by PCR and sequencing. The mutations delete most of the *ceh-19* homeobox and potential splicing together of the remaining two exons of transcript *ceh-19b* would cause a shift to an incorrect translational reading frame for the last exon. The truncated peptide encoded by such a hypothetical transcript would retain the potential acidic transcriptional activation domain but none of the homeodomain. Therefore, *tm452* and *tm461* are likely to be null alleles. The *tm452* allele was backcrossed to the N2 wild type seven times to generate UL3128, to remove other mutations that might be present in the original strain.

**FIG. 5:**
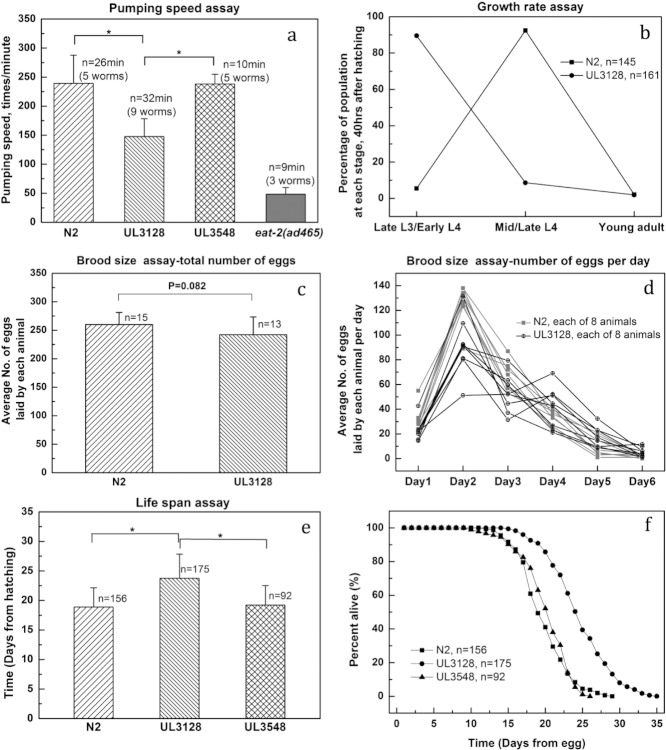
Effect of *ceh-19* deletion on life-history traits. (**a**) Mean pumping speeds ±s.d. are presented for N2, UL3128 (the backcrossed *ceh-19* mutant), UL3548 (the *ceh-19* rescued strain) and *eat-2 (ad465)* mutants. (**b**) The growth rates of N2 and UL3128 were compared under standard culturing conditions by recording the number of individuals that had become late L3/early L4s, mid/late L4s or young adults, 40 hrs after hatching. (**c**) UL3128 hermaphrodites appeared to produce slightly fewer progeny than N2 but the difference was not statistically significant (P=0.082) according to a one-way ANOVA. (**d**) Daily egg-laying of 8 UL3128 hermaphrodites was scored (black) and compared with that of 8 N2 hermaphrodites (grey) over the six-day reproductive period. (**e**) The average mean life span (with standard deviation) of N2, UL3128 and UL3548 individuals is plotted in days. (**f**) The percentage of individuals surviving on each day was determined for N2, UL3128 and UL3548. “*” indicates a statistically significant difference by a one-way ANOVA test, *P*<0.0001.

The original strains bearing the *tm452* and *tm461* alleles and UL3128 are viable and fertile and have no immediately obvious morphological, physiological, or locomotion defects. However, the specific expression of *ceh-19::gfp* in the pharyngeal motor neuron MC, which is necessary for rapid pharyngeal pumping (Avery and Horvitz, 1989), suggested the *ceh-19* mutants might be defective in pharynx function. Any such defect would need to be to a degree that is not strong enough to cause other more obvious defects, such as a starved body appearance. Closer examination of pumping behavior indicated that pharyngeal pumping rate of UL3128 was indeed reduced by more than 30% compared to N2 animals (Fig. 5a). In N2 individuals the pharynx pumped 239 ± 48.5 times per minute (mean ± s.d.) compared to 148 ± 30 times per minute for UL3128. The unbackcrossed *tm461* mutant animals have an almost identical pumping speed to UL3128 (data not shown), consistent with the two *ceh-19* alleles having essentially the same consequences. On an agar plate in the presence of abundant bacteria, pharynxes of N2 pump continuously at a near steady frequency. In contrast pharynxes of UL3128 pump in alternate cycles of 7-10 pumps at high and low frequencies, the slower-pumping phase responsible for reducing the average speed. This defect is not as severe as when both MC neurons were killed with a laser, which reduced the speed to 45 ± 6 pumps/minute (Raizen *et al*., 1995), indicating that MC function is not completely inactivated in the *ceh-19* mutants. The *ceh-19* mutant defect is not even as severe as that for *eat-2(ad465)* (Fig. 5a) which affects synapses between the pharyngeal muscles pm4 and pm5, post-synaptic to MC (Mckay *et al*., 2004). As for N2, pharyngeal pumping is absent in UL3128 animals on plates with no bacterial food, and when pumping is restored by supplementation with 10 mM serotonin UL3128 still pumps at a slower rate than N2. This suggests that the compromised pumping rate of UL3128 is due to a defect in the pharynx, and presumably MC itself, rather than in the ability to sense food. UL3128 was transformed with the fosmid fUL#HF004.1 bearing the full-length *ceh-19::gfp* translational fusion to rescue the *ceh-19* defect. The rescued strain, UL3548, had recovered pumping speed (241 ± 19.2 pumps/minute), very close to that of wild type worms, indicating that the pharynx defect in UL3128 is indeed due to the absence of *ceh-19* (Fig. 5a).

### Consequences of ceh-19(tm452) for Other Life-History Traits

Reduced food intake can influence other life-history parameters such as the development rate, and UL3128 animals showed slightly slower growth than N2, presumably as a result of the slower pharyngeal pumping. Growth rate was assessed in two ways. First, when two synchronized L1s were placed on each of six seeded agar plates, and incubated for 70 h at 20°C, 10 of 12 N2 worms had developed into mature adults, and there were scores of laid eggs with a few hatched L1 larvae on each plate. In contrast, when UL3128 L1s were used, each plate had only around ten eggs and no L1s had appeared. By the tenth day of incubation, the N2 populations had exhausted the bacteria but the UL3128 populations required another 20–24 h to use up the food source. In a second assessment, after 40 hr growth at 20°C, 3 of 145 synchronized N2 L1s had grown into young adults, 8 were late L3s/early L4s, and the remainder were mid/late L4s. For 161 UL3128 L1s, after 40 hr there were also 3 young adults, but the vast majority were late L3/early L4s and only 14 were mid/late L4s, in clear contrast with the N2s (Fig. 5b).

The period of fecundity is prolonged in UL3128 animals. Although the average number of embryos produced by each UL3128 hermaphrodite (brood size=242 ± 31, *n*=13) appeared slightly less than that of N2 (brood size=260 ± 21, *n*=15) (Fig. 5c) this difference was not found to be statistically significant. However, the period of fecundity of UL3128 seemed prolonged (Fig. 5d). N2 hermaphrodites laid most of their eggs (83.2 ± 4.1%, *n*=8) within the first 3 days of the reproductive period with a peak on the second day (Fig. 5d). UL3128 animals laid a smaller proportion of eggs during the first 3 days (72.1 ± 9.4%, *n*=8) and laid more eggs on subsequent days, especially on Day 4. In addition, UL3128 showed considerably more variation in individual egg-laying curves than N2. When the number of eggs retained in the uterus was checked at 90 h after hatching (on the 2nd day of the reproductive period), N2 adults retained on average 13 ± 4.6 (*n*=10, mean ± s.d.) eggs in the uterus and UL3128 had 8.7 ± 4.2 (*n*=10, mean ± s.d.). At 96 h after hatching, N2 had 9.5 ± 1.5 eggs within the uterus of each hermaphrodite (*n*=14, mean ± s.d.) and UL3128 had 8.2 ± 2.3 (*n*=13, mean ± s.d.). This provided further evidence of the slight difference of brood size and egg-laying pattern between N2 and UL3128.

UL3128 has an extended life span. Reduced food intake lengthens life span in *C. elegans* as observed for many *eat* mutants with their defects in pharyngeal function (Lakowski and Hekimi, 1998). UL3128 is also essentially a weak *eat* mutant and the life span assay for this strain was performed twice, independently (Fig. 5e,f). N2 worms on average lived 18.9 ± 3.3 days (*n*=156, mean ± s.d.), very close to the 19.5–21.6 days reported previously by Lakowski and Hekimi (1998) who also fed the worms on live OP50 bacteria. For UL3128 the lifespan was 23.8 ± 4.1 days (*n*=175, mean ± s.d.), a lifespan extension similar to that of *eat-1(e2343)* (23.9 ± 0.9 days, mean ± SEM) and *eat-6(ad997)* (23.6 ± 0.7 days, mean ± SEM) mutants, when assayed with the same protocol (Lakowski and Hekimi, 1998). The pharyngeal pumping speed of *eat-1(e2343)* mutants is not published, but the pharyngeal pumping rate of *eat-6 (ad997)* mutants (∼150 pumps/minute) (Doi and Iwasaki, 2008) was very close to that of UL3128. These observations are consistent with the good correlation between life span extension and the severity of the eating defect, as has been observed previously (Lakowski and Hekimi, 1998).

Rescue of *ceh-19* in UL3548 shortened lifespan compared to that of the *ceh-19* mutant, towards that of the wild type (Fig. 5e,f). The rescued animals had a lifespan of 19.2 ± 3.3 days (*n*=92, mean ± s.d.). Statistically, the UL3548 lifespan was significantly shorter than for UL3128 and was not significantly different from that for N2. Transformation with CEH-19 did appear to rescue the lifespan extension phenotype of the *ceh-19* mutant, as for the decreased pharyngeal pumping speed phenotype.

UL3128 animals enter into and exit from the dauer stage normally. Expression of *ceh-19* in ADF and PHA neurons might suggest a role connected with the dauer stage. Although UL3128 animals develop to adults at a slower rate, dauer formation was not observed in normal growth conditions; the *ceh-19* deletion does not confer a dauer formation constitutive (Daf-c) phenotype. Efficiency of dauer recovery for N2 and UL3128 was also compared and no difference was observed. All dauer animals (n=∼200) resumed development within 24 hrs after food became available again. Possible further roles of *ceh-19* in chemosensation, suggested from expression in these sensory neurons, were not investigated.

Finally, defecation did not appear affected in the *ceh-19* mutant. The period of the defecation cycle of UL3128 remained at 40 ± 1 s, similar to, but apparently even more regular than, N2 at 42 ± 3.8 s (mean ± s.d., *n*=5 worms each, 10 min of observation for each worm). This is consistent with previous observations that bacteria intake has only a minor effect on the period of the defecation cycle (Thomas, 1990).

### ceh-19 Is a Novel Eat Gene

Previous screens for pharyngeal pumping defects had yielded the Eat mutants (Avery, 1993). Almost all Eat mutations characterized so far result in severe impairment to the pharynx and most of them also affect tissues outside the pharynx (Avery, 1993; Shibata *et al*., 2000). The molecular nature of some of these genetically identified Eat genes, including *eat-1, eat-8, eat-9, eat-10, eat-13, eat-14, eat-15*, and *eat-17*, remain to be determined. Among these, *eat-1* and *eat-10* have been mapped onto linkage group IV where *ceh-19* is found. *eat-10(ad606)* is on the left arm of LGIV (IV: −26.74 ± 0.306cM), far from *ceh-19* (IV:3.51 ± 0.001cM). However, *eat-1* was mapped onto LGIV at 4.8 ± 6.395cM, and both its recessive alleles, *ad427* and *e2343*, have slow and irregular pharyngeal pumping, like *tm452*. The very large confidence interval of the *eat-1* location presumably reflects problems with the genetic mapping but does cover the locus of *ceh-19*. We found, however, that all three *eat-1* mutants have even slower pumping speed than the *ceh-19* deletion mutants: DA531[*eat-1(ad427)IV*] at 89.1 ± 21.7, CB4394 [*eat-1(e2343) unc-31(e928)IV*] at 87 ± 17.9, and DA449[*eat-1(e2343) dpy-20(e1282)IV*] at 106 ± 17.4 times per minute, respectively (mean ± s.d, n=10). And direct tests revealed *ceh-19* and *eat-1* are not allelic; PCR amplification of *ceh-19* from genomic DNA of *eat-1* mutants yielded products of wild type sizes and *tm452* complements genetically all three *eat-1* mutant alleles. Therefore, the molecular nature of *eat-1* remains to be determined and *ceh-19* is a newly identified *Eat* gene, a homeobox gene that is necessary for *C. elegans* pharynx development and proper pumping.

### UL3128 Animals Have Morphological Defects in MC

To assess whether the specification and development of MC, ADF and PHA were affected by the *ceh-19* deletion and also whether CEH-19 is required for its own expression in these neurons, their morphology was examined in the *ceh-19(tm452)* deletion background using *ceh-19b^prom^::gfp* fusions. The chromosomally integrated transgenic array *leIs2703[ceh-19b^prom^*::*gfp, unc-119(+)]* was crossed from strain UL2703 into the *ceh-19(tm452)* mutant background of UL3128 to give strain UL3413. The extrachromosomal array *leEx3011* and *leEx3012* both carrying the recombineered fosmid fUL#HF005.1 with the *ceh-19b* protein coding region replaced with *gfp*, in the strains UL3011 and UL3012 respectively, were also crossed into the *ceh-19(tm452)* mutant background of UL3128 to give strains UL3013, and UL3019 and UL3022.

**FIG. 6:**
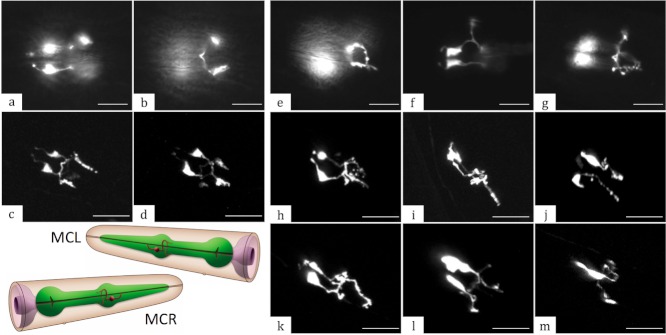
The *ceh-19* deletion causes axonal defects in MC neurons. In the wild type, MCs have symmetrically located cell bodies and well-organized, consistent axonal projections, as observed by both epifluorescence (**a, b**) and confocal (**c, d**) microscopy. In the *ceh-19(tm452)* mutant background, the *ceh-19b::gfp* transcriptional reporter fusions were still expressed in the MCs, but revealed various subtle to moderate axonal defects upon epifluorescence (**e–g**) and confocal microscopy (**h–m**). All images were captured in the region of anterior pharyngeal bulb with anterior towards the upper left. Strains presented are: UL3012 (**a–d**), UL3022 (**e–g**), UL3013 (**h–i**), UL3019 (**j**) and UL3022 (**k–m**). A panel depicting the morphology of MCL and MCR (red) with respect to the pharynx (green) in the wild type is included for comparison (from WormAtlas). Scale bars represent 10 µm in all panels.

In each strain with the *ceh-19(tm452)* deletion background, GFP expression in all three pairs of neurons remained at a similar level to that in the wild type background, suggesting MC, ADF, and PHA are still born normally in the absence of CEH-19, and *ceh-19* expression is not self-regulated. The *ceh-19b^prom^*::*gfp* expression was also examined in dauer animals in the *ceh-19* mutant background as the change in strength of expression in ADF in this stage might have depended on functional CEH-19, but again no difference from wild type levels was observed (data not shown). Although apparently lacking a role in the fundamental generation of these nerve cells, CEH-19 could still be functional in their specific terminal differentiation. Indeed, in 90% (n>100) of mutant animals, there were axonal defects in the MC neurons to varying degrees. MCs in wild type animals have symmetrical, well-organized cell bodies and consistent axonal projections as revealed under epifluorescence microscopy (Fig. 6a, b) and in confocal microscopy sections (Fig. 6c, d). Although usually both MC cell bodies were still present in the *ceh-19* mutant background, they were often different sizes and shapes (Fig. 6h-l). Moreover, severe abnormalities were observed for MC axons, by both epifluorescence and confocal microscopy (Fig. 6e-m), which presumably would result in abnormal inter-neuronal and neuro-muscular connections. Occasionally, in the mutant background, GFP was only apparent in one MC (Fig. 6m). These defects are consistent with the moderate pharyngeal pumping defect observed for the *ceh-19* mutant animals.

No axonal defects were observed by epifluorescence microscopy for ADF and PHA neurons in the *ceh-19* deletion background (data not shown). These amphid and phasmid neurons might have minor structural and/or synaptic defects beyond detection from observations of the GFP expression but which could be revealed by electron microscopy.

### Regulatory Context of ceh-19 in MC

**FIG. 7:**
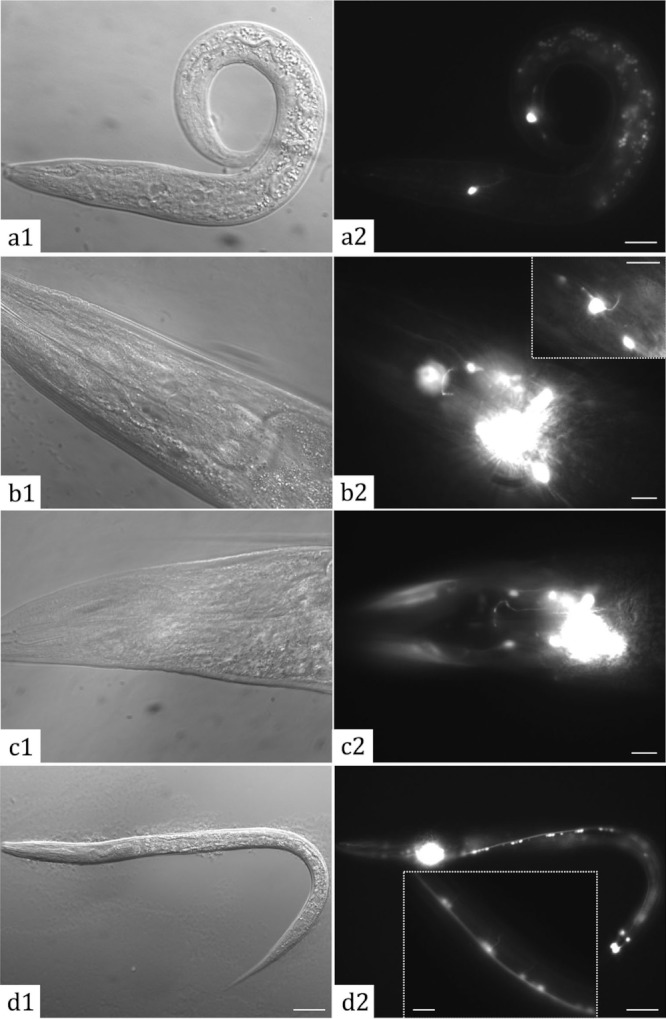
Regulation upstream and downstream of *ceh-19*. Larva hatched from UL2703 animals transgenic for *ceh-19b^prom^::gfp* and subject to RNAi for *pha-4* are pharynxless (**a1**), and although expression in ADF and PHA was maintained, expression in MC was lost (**a2**). In a wild type background, *flp-2^prom^::gfp* is expressed in MC (**b2** and **b2** inset) and other neurons (**b1/2**). In the *ceh-19(tm452*) background, *flp-2^prom^::gfp* expression was not detected in MCs but was still present in other neurons at the same level as in wild type (**c1/2**). *flp-2^prom^::gfp* expression was observed in ventral cord motor neurons in the dauer stage only (**d1/2**) including the axon (**d2** inset), here in the wild type. Bars represent 10 µm in **a–c**, **b2** inset and **d2** inset, and 25 µm in **d**. Corresponding images were captured by DIC (**a–d1**) or epifluorescence (**a–d2**) microscopy.

PHA-4 is the pharyngeal master regulator required for development of all cells of the pharynx (Mango, 2009) and the expression of *ceh-19* in MC is dependent on this transcription factor. We assayed the dependence of *ceh-19* expression on PHA-4 by applying *pha-4* RNAi to gravid UL2703, with the chromosomally integrated *ceh-19b^prom^::gfp* fusion, and examining GFP in the progeny. As expected, knocking down PHA-4 resulted in embryonic lethality and/or larval arrest for UL2703. Animals that did manage to hatch out appeared to lack a pharynx with no GFP corresponding to MC, although GFP in ADF and PHA remained (Fig. 7a1/2). Presumably no cell with an MC fate is generated with PHA-4 absent.

CEH-19 is required for *flp-2* expression in MC*. flp-2* and *flp-21* both encode FMRFamide-like peptide neurotransmitters and have been reported to be expressed in MC, in addition to multiple other nerve cells (Kim and Li, 2004). To assay the genetic dependence of these genes on CEH-19, we generated strains UL3795 and UL3793, transgenic for *flp-2^prom^::gfp* and *flp-21^prom^::gfp* fusions respectively, and indeed detected GFP expression in MC neurons in the wild type background. The reporter fusions were then crossed into UL3128 with the *ceh-19* deletion background, resulting in strains UL3881 [*ceh-19(tm452); flp-2^prom^::gfp*] and UL3891 [*ceh-19(tm452); flp-21^prom^::gfp*], to assess their regulation by CEH-19. While there was no difference in MC expression of *flp-21^prom^::gfp* between the wild type and *ceh-19* mutant (data not shown), *flp-2* expression in MC appeared dependent on CEH-19. In the wild type background, *flp-2^prom^::gfp* gave a clear consistent GFP signal in MCs, in UL3795 and 3 other independent transgenic strains, although expression was normally weaker than in other cells (Fig. 7b1/2). In contrast, in the *ceh-19* mutant, *flp-2^prom^::gfp* expression in MC was not detectable (Fig. 7c1/2), suggesting CEH-19 is required for *flp-2* expression in MC. This requirement was specific to MC, the only cells in which *ceh-19* and *flp-2* expression overlaps, with *flp-2* expression in other cells not affected in the more than 50 individuals where expression was carefully and specifically examined in this regard. MC expression of *flp-2^prom^::gfp* in dauer animals in both the wild type and *ceh-19* mutant backgrounds was also compared. Although *ceh-19b^prom^::gfp* was expressed strongly in MC during the dauer stage, MC expression of *flp-2^prom^::gfp* in dauers with wild type background was weaker than in non-dauers or even absent. Nevertheless *flp-2^prom^::gfp* MC expression was never observed in the *ceh-19* mutant dauers, confirming the requirement of CEH-19 for *flp-2* expression, even in the dauer. Interestingly, while examining *flp-2^prom^::gfp* MC expression, a strong GFP signal was observed in ventral cord motor neurons of dauers in both wild type and *ceh-19* mutant backgrounds (Fig. 7d1/2). Such expression for *flp-2* was not reported previously and was not seen in non-dauers, indicating a potential involvement of *flp-2* in dauer physiology.

Evidence for direct interaction between PHA-4 and *ceh-19* and between CEH-19 and *flp-2* were sought using the yeast one hybrid (Y1H) approach. The strategy could also have yielded additional targets of CEH-19 and regulators of *ceh-19*. A previously described *C. elegans* transcription factor yeast array (Reece-Hoyes *et al*., 2011) was used in a yeast-one-hybrid screen with the *ceh-19b* promoter as bait. Out of the 755 transcription factors in the array, three, TBX-8, TBX-9, and MLS-2, appeared to bind the *ceh-19b* promoter. Although genetically required for *ceh-19* expression in MC, PHA-4 did not appear to bind the *ceh-19* promoter in these Y1H TF array screens. This was also specifically tested with *ceh-19b^prom^-lacZ* bait yeasts directly transformed with the Gal4AD-PHA-4 and Gal4AD-only prey plasmids. Both showed the same level of *lacZ* expression, yielding no support for specific binding of PHA-4 to the *ceh-19b* promoter. Twenty genes reported to be expressed in MC, ADF and/or PHA, including *flp-2* and two transcription factor genes, *lin-11* and *lim-4*, were selected as potential CEH-19 targets (Supplementary Table 2). Promoters for these genes, either retrieved from the Promoterome or cloned *ab initio*, were fused with the Y1H *lacZ* and *His3* reporters for screening as baits versus the *C. elegans* TF yeast array. CEH-19 did not appear to bind to any of these baits, although various other transcription factors did (data not shown).

## DISCUSSION

The *C. elegans* genome encodes ∼100 homeodomain transcription factors, 24 of which lack any other highly conserved motif (Reece-Hoyes *et al*., 2005). Currently these “homeodomain-only” transcription factors, including *ceh-19*, are less well characterized although over half are essential, with various developmental and behavioral roles. Reporter gene fusions have been assayed for most and typically revealed expression in a range of cell types, e.g., (Reece-Hoyes *et al*., 2007). The restricted distribution of reporter expression for *ceh-19*, however, led to its study here.

The lack of reporter expression with a *ceh-19a* specific fusion, and lack of additional components for the fusion targeting both transcripts as compared to fusions targeting *ceh-19b* specifically, suggests *ceh-19a* is at best poorly expressed under laboratory conditions. The sequence of the donor splicing sites of *ceh-19b* intron 1 (AGgtaatcat) matches more closely to the well-respected *C. elegans* consensus sequence (AGgtaagttt) than does that of *ceh-19a* intron 1 (TTgtatgaaa). Furthermore, the predicted CEH-19a protein would lack the region probably responsible for transcriptional activation in CEH-19b. The significance of *ceh-19a*, therefore, looks doubtful and the *ceh-19b* gene model appears to represent the full function of the gene.

Although the defect in pharyngeal pumping in the *ceh-19* mutant is likely to relate purely to expression in MC, the clear expression of the *ceh-19::gfp* fusion in ADF and PHA suggests *ceh-19* contributes to the sensory roles of these neurons. Defects relating to ADF and PHA sensory function may, however, be subtle due to redundancy with other cells or genes, or may not have been apparent because of ecological specificity. Nevertheless, the striking up-regulation in the ADF of dauers could be significant in this regard. *C. elegans* dauer entry and exit involve integration and transformation of environmental cues (dauer pheromone, nutrients, and temperature) into endocrine signals by amphid neurons. Amphids ADF, ASI and perhaps ASG act redundantly to prevent dauer formation in favourable conditions, and amphid ASJ is critical to exit from the dauer stage with some minor contributions from ADF or ASI, or ASG (Bargmann and Horvitz, 1991a; Bargmann and Horvitz, 1991b). This redundancy amongst the amphids means the lack of an effect of the *ceh-19* deletion on dauer entry or exit is not surprising. The up-regulation suggests increased levels of CEH-19 are important in preparing ADF for a role in the dauer stage, presumably for sensing of conditions for dauer exit or perhaps contributing to the resilience to adverse conditions characteristic of the dauer stage, in this case specifically of ADF. Such roles may only be revealed with ASI, ASG and ASJ inactivated. The increased *ceh-19* expression in ADF would itself be a consequence of endocrine signals acting to direct dauer entry. This, along with the *flp-2::gfp* expression in ventral cord neurons of only the dauer stage, and the expression of this reporter fusion in MC being *ceh-19* dependent seems unlikely to be coincidental but the biological significance is not yet apparent. *ceh-19* has not been identified previously in global expression analyses seeking genes expressed specifically or with altered levels in the dauer stage (Holt, 2006; Jones *et al*., 2001; Wang and Kim, 2003). This may be because the problem of low expression, as seen with other transcription factor genes, is further exacerbated by expression being restricted to just a few small cells. Reporter gene fusions provide the localized sensitivity needed to observe such subtle effects.

The minor nature of the *ceh-19* mutant's defects, compared with those upon MC ablation, suggests MC function is only partially disrupted. As MC cell processes are abnormal in *ceh-19(tm452)* mutants, a simple hypothesis would be that receipt or transmission of neural signaling is impaired due to the improperly specified synaptic connections between MC and muscles and/or other neurons. As *tm452* and *tm461* are likely to be null alleles, *ceh-19* may only be required for partial or non-essential aspects of MC specification, with only limited types of terminal differentiation genes being controlled by CEH-19. Alternatively, the robustness of transcription factor regulatory networks may mean that the level of expression of genes expressed for MC terminal differentiation are only moderately perturbed by the complete absence of CEH-19 activity. CEH-19 would contribute to the level of expression of many genes needed for MC fate, but other transcription factors are required for MC differentiation, with roles that remain to be identified, and buffer against *ceh-19* loss.

The gene *flp-2*, the identified target of CEH-19 regulation in MC, encodes two FMRFamide-like neuropeptides (FLPs), FLP-2A and FLP-2B. Different *C. elegans* pharyngeal neurons express different sets of modulatory FLP neuropeptides (Husson *et al*., 2007; Kim and Li, 2004). While eleven *flp* genes encode inhibitors of pharyngeal activity, eight encode excitatory peptides (Papaioannou *et al*., 2005). FLP-2A, expressed in pharyngeal neurons M4, MC and I5, enhances pharyngeal activity. In contrast, FLP-21, also expressed in MC, suppresses pumping rate (Papaioannou *et al*., 2005). Hence, in a single neuron, MC, at least two FLPs are expressed with opposing activity, acting in response to different environmental stimuli to fine-tune pumping rate. Presumably, complex transcription factor regulatory networks integrate the subtly distinct nerve cell fates with the different environmental situations encountered so as to achieve the expression of each member of the range of *flp* genes precisely where, when, and to the levels required. CEH-19 is essential for *flp-2* expression in MC. However, interruption of *flp-2* by RNAi caused embryonic lethality, slow growth and larval arrest (Simmer *et al*., 2003), a more severe phenotype than the slow pumping when only inactivated in MC and in accordance with its broad expression and a wider role. Clearly other transcription factors must input into *flp-2* such that expression of *flp-2* doesn't occur wherever CEH-19 is expressed and can occur in cells beyond where CEH-19 is expressed. As the MC fate is only partially perturbed in *ceh-19* mutants, other transcription factors must control other MC terminal differentiation genes, such as *flp-21*. Furthermore, transcriptional control of *flp-2* is not the only role for CEH-19 in MC. The MC morphological defects in *ceh-19* mutants indicate transcription of other genes is needed for proper MC differentiation.

Despite the genetic evidence of regulatory dependency, CEH-19 did not bind the *flp-2* promoter and PHA-4 did not bind the *ceh-19* promoter, in Y1H assays. These regulatory interactions could be indirect *in vivo* with other intermediate transcription factors, or direct but with co-factors required. A direct interaction of PHA-4 to the *ceh-19b* promoter might have been expected as PHA-4 directly activates many genes expressed in the different cell types in the pharynx (Gaudet and Mango, 2002). Furthermore, modENCODE ChIP experiments identified PHA-4 binding to the *ceh-19* promoter, within the region assayed in the Y1H experiments, but only in L1s and not other stages (Gerstein *et al*., 2010). Given the lack of any other supporting data, the significance of our finding of TBX-8, TBX-9, and MLS-2 binding to the *ceh-19* promoter in Y1H assays, for regulation of *ceh-19* expression *in vivo* is unclear. Although our targeted Y1H screen for potential CEH-19 targets, using 20 candidate gene promoters, was unsuccessful, a larger-scale screen has since revealed Y1H binding of CEH-19 to promoters of *cog-1*, *vha-15*, *hlh-15* and *lbp-8* (Reece-Hoyes *et* *al*., 2011). The biological relevance of these interactions for *ceh-19* function *in* *vivo* is also as yet unclear. Genome-wide screens for direct protein::DNA interactions involving transcription factor combinations would be a huge undertaking even with an assay as easy to apply as the Y1H. Other data, such as from expression pattern determinations or ChIP, may be needed to reduce the number of transcription factors that need to be tested, combinatorially, in such assays before the details of transcription factor regulatory networks can be revealed.

## METHODS

### *C. elegans* Strains and Mutant Alleles

All strains were maintained at 20°C on 5 cm NGM agar plates seeded with *E. coli* OP50 as food source (Sulston and Hodgkin, 1988). *C. elegans* N2 (Bristol) was used as wild type. Transgenic strains are UL2701 [*unc-119(ed3)III; leEx2701(ceh-19b^Prom^::gfp, unc-119(+))*], UL2702 [*unc-119(ed3)III; leIs2702 (ceh-19b^Prom^::gfp, unc-119(+))],* UL2703 [*unc-119(ed3)III; leIs2703(ceh-19b^Prom^::gfp, unc-119(+))*] (Reece-Hoyes *et al*., 2007). *C. elegans* strains with *ceh-19(tm452)* and *ceh-19(tm461)* alleles were from the Mitani Laboratory (http://www.shigen.nig.ac.jp). The *ceh-19(tm452)* allele was backcrossed into N2 seven times to generate the strain UL3128, which was used in most phenotypic assays.

Transgenic strains generated in this work include: UL3010 [*leEx3010(fUL#HF005.1, rol-6(su1006))*], UL3011 [*leEx3011(fUL#HF005.1, rol-6(su1006))*], and UL3012 [*leEx3012(fUL#HF005.1, rol-6(su1006))*], each carrying the recombineered fosmid fusion fUL#HF005.1 in an extrachromosomal array; UL3014 [*leEx3014(fUL#HF004.1, rol-6(su1006))*] and UL3308 [*leEx3308(fUL#HF004.1, rol-6(su1006))*], each carrying the recombineered fosmid fusion fUL#HF004.1 in an extrachromosomal array; UL3413 [*ceh-19(tm452)IV; leIs2703(ceh-19b^Prom^::gfp, unc-119(+))*], created by crossing *leIs2703* from UL2703 into UL3128; UL3013 [*ceh-19(tm452)IV; leEx3011(fUL#HF005.1, rol-6(su1006))*], created by crossing *leEx3011* from UL3011 into UL3128; UL3019 [*ceh-19(tm452)IV; leEx3012(fUL#HF005.1, rol-6(su1006))*] and UL3022 [*ceh-19(tm452)IV; leEx3012(fUL#HF005.1, rol-6(su1006))*], created by crossing *leEx3012* from UL3012 into UL3128; UL3548 [*ceh-19(tm452)IV; leEx3014(fUL#HF004.1, rol-6(su1006))*], created by crossing *leEx3014* from UL3014 into UL3128; UL3795 [*leEx3795(flp-2^prom^::gfp, rol-6(su1006))*], UL3793 [*leEx3793(flp-21^prom^::gfp, rol-6(su1006))*] and UL3891 [*ceh-19(tm452)IV; leEx3891(flp-21^prom^::gfp, rol-6(su1006))*], created by microinjection of the wild type, N2, or the *ceh-19* mutant, UL3128, with the plasmid containing the *flp-2^prom^::gfp or flp-21^prom^::gfp* fusions constructed by Gateway recombination; UL3881 [*ceh-19(tm452)IV; leEx3795(flp-2^prom^::gfp, rol-6(su1006))*], created by crossing *leEx3795* from UL3795 into UL3128.

### Reporter Gene Fusions and Expression Pattern Analysis

Recombineering of *C. elegans* fosmid clones was carried out according to Bamps and Hope (2008). *flp-2^prom^::gfp* and *flp-21^prom^::gfp* fusions were generated by Gateway recombinational cloning as described by Reece-Hoyes *et al*. (2007). Primer sequences are provided in Supporting Information Table 1.

A Leica DMR HC microscope fitted with GFP (Chroma 41017), YFP (Chroma 51017), and DAPI/FITC/TexasRed (Chroma 61002) filter sets was used to observe the reporter expression patterns and to identify neuronal identities by DIC. A Hamamatsu ORCA-ER B/W CCD camera was used to capture images with an Improvision Openlab image processing system. A Zeiss LSM 510 confocal system was used to capture z-stacks at 1 µm intervals. Improvision Volocity was used to visualize, organize and export images from Openlab and the confocal system.

### Behavioral Assays

Pharyngeal pumping speed was measured by direct inspection at 160× magnification at room temperature. Individuals, 5 per strain, were picked to a fresh NGM plate with OP50 and allowed to settle at room temperature for 10 minutes before counting pharyngeal pumps during 5 periods of 1 minute.

The defecation motor cycle of well-fed, healthy adults was measured manually under 100× magnification at room temperature. Only the posterior body wall muscle contraction (pBoc) and expulsion muscle contraction were followed. The time of each expulsion was recorded over two 10 min windows for each of five individuals per strain.

Brood size was measured by picking L4 animals individually to fresh culture plates, with subsequent serial transfer at 24-hr intervals. The fertilized eggs laid in each 24-hr period were counted until no more eggs were laid. The total number of eggs laid by each hermaphrodite was taken as the brood size.

To compare growth rates, synchronized L1s, hatched overnight on culture plates without bacterial food, were transferred onto a fresh 5 cm NGM plate with an OP50 bacterial lawn grown from 500 μl of an overnight culture and maintained at 20°C. Their development was monitored every day, recording on which day all the bacteria were consumed. At 40 hours after L1s had resumed development upon supply of bacterial food, the numbers of animals in late L3/early L4, late L4, and young adult stage were counted.

To measure life span, about 200 L1s hatched out during a 2-h period were left to grow to the L4 stage on culture plates at 20°C. These L4s were then distributed to fresh seeded plates, 10 per plate. Individuals were transferred to new plates every day during their reproductive period and then examined every day until their death, as determined by lack of movement even in response to physical stimulation. Each day, dead individuals were removed from the plates and the deaths were recorded.

Statistics analyses of the behavioral assays were performed using one-way ANOVA in OriginPro7.5 (Origin Lab Corporation).

### RNAi by Feeding

RNAi by feeding was carried out as described by (Kamath and Ahringer, 2003). Standard NGM plates were supplemented with ampicillin (50 µg/ml), tetracycline (10 µg/ml), and IPTG (isopropyl β-D-1-thiogalactopyranoside) (1 mM), and seeded with bacteria, from the RNAi library from Geneservice (Kamath and Ahringer, 2003), verified by restriction enzyme digestion of purified plasmids. A few L3-L4 hermaphrodites of strains transgenic for a *gfp* reporter fusion, were transferred from an area off of the bacterial lawn of an OP50 seeded NGM plate, first to an unseeded NGM plate for a few minutes, and then to the RNAi plates. The RNAi plates were maintained at 20°C for 3 days before observing the progeny. The negative control was HT115 bacteria containing pL4440, as for clones in the RNAi library, but without an insert between the T7 promoters. An equivalent bacterial strain with a pL4440 insert for *unc-22* was used as a positive control.

### Yeast One-Hybrid Screens

Yeast one-hybrid (Y1H) screens and assays were performed as described previously (Deplancke *et al*., 2004; Reece-Hoyes *et al*., 2011; Vermeirssen *et al*., 2007; Walhout, 2006). Promoter entry clones were either retrieved from the *C. elegans* Promoterome library or generated *ab initio* by Gateway cloning. (Primers are listed in Supplementary Table 1.) *Promoter::reporter (HIS3/lacZ)* bait fusions were generated by Gateway LR recombination reactions between the promoter entry clones and the *pMW2-HIS3* and *pMW3-lacZ* yeast expression vectors. *Promoter::reporter* bait fusions were linearized and used in transformation of the YM4271 (MATa) yeast host strain with integration into the genome by homologous recombination. Twelve clones from each integration were assayed for self-activation of *HIS3* and *lacZ* expression and the clone with the lowest level for each bait was used for subsequent screens.

Screens for transcription factors that interact with a promoter (Y1H) bait were performed using the enhanced Y1H (eY1H) approach (Reece-Hoyes *et al*., 2011). eY1H screens are more efficient than traditional library screens because the transcription factors are presented to the baits as an array of yeast prey strains (Yα1867, MATα), each transformed with a different Gal4AD-TF fusion. 755 *C. elegans* transcription factors were included in the array, with each TF represented four times and thus retested inherently. A bench top robot (RoToR, Singer Instrument, Somerset, UK) was used to precisely transfer the (up to) 1536 yeast colonies present on each media plate. To prepare for screens, bait yeast cells were propagated as lawn cultures in standard RoToR dishes containing YAPD media. The transcription factor array was maintained on Sc-Trp media in sets of three 1536-colony plates each containing four copies of up to 384 AD-TF prey yeast clones. To set up a mating, each bait lawn and the transcription factor array were sequentially copied to YAPD plates. The yeasts were grown at 30°C for 3 days before being copied to Sc-His-Ura-Trp plates to select for successfully mated diploids. Diploids were grown for two days before being copied to Sc-His-Ura-Trp plus 5 mM 3-AT (3-amino-1,2,4-triazole) and 80 mg/ml X-gal (5-bromo-4-chloro-3-indolyl-β-D-galactoside) plates and incubated at 30°C. Plates were monitored over the next 5–7 days for the expression level of the reporters. Colonies that can grow in the absence of histidine, overcome the inhibitory effects of 3-AT, and turn X-gal into a blue compound are expressing the reporters, indicating a transcription factor-promoter (Y1H) interaction. Positives, with at least two of the four colonies containing a particular transcription factor showing reporter expression, were identified according to their array coordinates. The transcription factor ORFs of positive colonies were PCR amplified using primers corresponding to the vector and sequenced to verify the identity of the transcription factors.

To directly test individual transcription factor - promoter (Y1H) interactions, plasmids encoding Gal4AD-TF prey fusions were transformed into haploid promoter::reporter bait strains and activation of reporters in transformants was assessed with a β-galactosidase assay on overlay filter membranes and from growth on selective plates plus 20, 40, or 60 mM 3-AT (Vermeirssen *et al*., 2007).
