# Supplementary material for: The Caenorhabditis elegans Homeobox Gene ceh-19 Is Required for MC Motorneuron Function
Source: Genesis. 2013 Jan 12;51(3):163–78. doi: 10.1002/dvg.22365 (PMC3638342; doi:10.1002/dvg.22365)
Supplement: Supplementary file 1 [file dvg0051-0163-SD1.docx]

**Supplementary Table 1. PCR primers**

| **Usage** | **Name** | **Sequences*** |
| --- | --- | --- |
| recombineering *gfp* fusions | CEH-19-GFP-For (after ATG) | ttctcaactctacattttcttaaagtcttttaaataaaaactattgaaaaATGAGTAAAGGAGAAGAACT |
|  | CEH-19-GFP-For (before STOP) | cacctacaactcttgcctgtcacgtcaactccctgttcgcttgtgagcaaATGAGTAAAGGAGAAGAACT |
|  | CEH-19-GFP-Rev (before STOP) | tgcaatagaattctcaatgtaatataaatttcataaacatgaggaagctaTTTGTATAGTTCATCCATGC |
| *ceh-19a* Gateway cloning | attB4-ceh-19aprom-For | GGGGACAACTTTGTATAGAAAAGTTGTAtctaacctgaaaatattgat |
|  | attB1r-ceh-19aprom-Rev | GGGGACTGCTTTTTTGTACAAACTTGTCatgttatagaggtgtgaata |

*For recombineering primers, for fusions at the start (after ATG) or end (before STOP) of the *ceh-19* protein-coding region, the gene-specific sections are in lower case and those corresponding to the ends of the *gfp* reporter are in upper case, for priming in the forward (For) or reverse (Rev) direction with respect to transcription. For the Gateway cloning primers, the gene specific sections are in lower case and the *att* recombination site sections are in upper case.
