# Supplementary material for: The Caenorhabditis elegans Homeobox Gene ceh-19 Is Required for MC Motorneuron Function
Source: Genesis. 2013 Jan 12;51(3):163–78. doi: 10.1002/dvg.22365 (PMC3638342; doi:10.1002/dvg.22365)
Supplement: Supplementary file 2 [file dvg0051-0163-SD2.docx]

**Supplementary Table 2. Promoter baits for Y1H screens**

| **Relevant expression sites** | **Genes** | ***Entry clones** | **^†^*Prom::HIS3/***  ***lacZ* fusions and**  **yeast strains** | ^‡^ **S-A**  **level** | ^$^ **Ref.** | **Promoter sizes,**  **start-end sequences** |
| --- | --- | --- | --- | --- | --- | --- |
| MC, ADF,  PHA | *F20D12.6b/ceh-19b* | Promoterome | WL/JRH091 | L | 1 | 1500, gcgaaaaaca-ttgaaaaATG |
| MC | *C52A11.4/mpz-1* | pUL#HF019 | HF-P_mpz-1 | H | 4 | 3669, gtacgcacct-tcccatggtg |
|  | *F01E11.5c/tyra-2c* | pUL#HF017 | HF-P_tyra-2c | M | 5 | 3001, tgtcatccgg-catcaagATG |
|  | *C26F1.10/flp-21* | pUL#HF022 | HF-P_flp-21 | L | 6, 7 | 3038, tgcgtctcgt-tcagacgATG |
|  | *W07E11.3/flp-2* | pUL#HF008 | HF-P_flp-2 | H | 6 | 3000, tcattcattt-gcaaaccATG |
| ADF | *R102.2* | pUL#HF020 | HF-P_R102.2 | L | 8 | 588, tttgatttgc-actagccATG |
|  | *K10G6.4* | pUL#HF015 | HF-P_K10G6.4 | L | 8 | 1321, ttttcaatga-gtgaaaaATG |
|  | *Y102E9.1a.4/odr-4* | pUL#HF021 | HF-P_odr-4 | L | 12 | 1936, tggaagggag-agaatccATG |
|  | *M28.7/nph-1* | Promoterome | HF-P_M28.7 | H | 8 | 351, aatttatttt-aagcaggATG |
|  | *R13H4.1/nph-4* | Promoterome | HF-P_nph-4 | L | 8 | 2000, atgactcata-tcgaaaaATG |
|  | *C55H1.2/gpa-10* | Promoterome | WL/Y1H038 | M | 11 | 2000, ctggtcgaaa-gggaaacATG |
|  | *C34D1.3/odr-3* | Promoterome | WL/Y1H043 | L | 11 | 2000, ttcagcaagt-tttagatATG |
|  | *ZC64.4/lim-4* | Walhout Lab | WL/Y1H019 | L | 14 | 1997, tgacacgatt-aagttgcacc |
|  | *B0212.5/osm-9* | Walhout Lab | WL/Y1H044 | L | 15 | Not available |
|  | *W01C8.6/cat-1* | pUL#HF023 | HF-P_cat-1 | M | 9 | 3095, tcacgctcta-aggaggtATG |
| ADF, PHA | *C02H7.1/dyf-11* | pUL#HF024 | HF-P_dyf-11 | L | 10 | 3194, ggatgtcgtg-aagtgcgATG |
|  | *E02C12.5/gpa-3* | pUL#HF009 | HF-P_gpa-3 | M | 11 | 3014, gcaggatttc-aacaattATG |
|  | *F18E2.5/gpa-13* | pUL#HF016 | HF-P_gpa-13 | M | 11 | 2726, gcagagtaga-aattgcaATG |
|  | *C33A12.4* | Promoterome | HF-P_C33A12.4 | M | 8 | 579, cactggcttt-gacaacaATG |
|  | *K07C11.10* | Promoterome | HF-P_K07C11.10 | M | 8 | 1619, tctatctcaa-attcgaaATG |
|  | *ZC247.3/lin-11* | Walhout Lab | WL/Y1H020 | L | 13 | 1997, tcactgaaaa-tcccttctca |

* **Promoter** **Entry clones** were either obtained from the *C. elegans* Promoterome, or generated in this work as indicated by the plasmid name pUL#HFxxx. ^†^ **Yeast expression fusions and strains** were either obtained from the Walhout Laboratory (WL/xxx) or generated in this work (HF-P-xxx). ^‡^ **S-A level** refers to Self-Activation level of the yeast strains (L-Low, M-Medium, and H-High). ^$^ **Reference:** 1. This work; 2. (Cassata *et al.* 2000); 3. (Kage *et al.* 2005); 4. (Xiao *et al.* 2006); 5. (Rex *et al.* 2005); 6. (Kim and Li 2004); 7. (Rogers *et al.* 2003); 8. (Kunitomo *et al.* 2005); 9. (Duerr *et al.* 1999); 10. (Kunitomo and Iino 2008); 11. (Jansen *et al.* 1999); 12. (de Bono *et al.* 2002); 13. (Hobert *et al.* 1998); 14. (Sagasti *et al.* 1999); 15. (Colbert *et al.* 1997); 16. (Troemel *et al.* 1995)

References

Cassata, G., H. Kagoshima, Y. Andachi, Y. Kohara, M. B. Durrenberger *et al.*, 2000 The LIM homeobox gene *ceh-14* confers thermosensory function to the AFD neurons in *Caenorhabditis elegans*. Neuron **25:** 587-597.

Colbert, H. A., T. L. Smith and C. I. Bargmann, 1997 OSM-9, a novel protein with structural similarity to channels, is required for olfaction, mechanosensation, and olfactory adaptation in *Caenorhabditis elegans*. J Neurosci **17:** 8259-8269.

de Bono, M., D. M. Tobin, M. W. Davis, L. Avery and C. I. Bargmann, 2002 Social feeding in *Caenorhabditis elegans* is induced by neurons that detect aversive stimuli. Nature **419:** 899-903.

Duerr, J. S., D. L. Frisby, J. Gaskin, A. Duke, K. Asermely *et al.*, 1999 The *cat-1* gene of *Caenorhabditis elegans* encodes a vesicular monoamine transporter required for specific monoamine-dependent behaviors. J. Neurosci. **19:** 72-84.

Hobert, O., T. D'Alberti, Y. Liu and G. Ruvkun, 1998 Control of neural development and function in a thermoregulatory network by the LIM homeobox gene *lin-11*. J. Neurosci. **18:** 2084-2096.

Jansen, G., K. L. Thijssen, P. Werner, M. van der Horst, E. Hazendonk *et al.*, 1999 The complete family of genes encoding G proteins of *Caenorhabditis elegans*. Nature Genetics **21:** 414-419.

Kage, E., Y. Hayashi, H. Takeuchi, T. Hirotsu, H. Kunitomo *et al.*, 2005 MBR-1, a Novel Helix-Turn-Helix transcription factor, is required for pruning excessive neurites in *Caenorhabditis elegans*. Curr. Biol. **15:** 1554-1559.

Kim, K., and C. Li, 2004 Expression and regulation of an FMRFamide-related neuropeptide gene family in *Caenorhabditis elegans*. The Journal of Comparative Neurology **475:** 540-550.

Kunitomo, H., and Y. Iino, 2008 *Caenorhabditis elegans* DYF-11, an orthologue of mammalian Traf3ip1/MIP-T3, is required for sensory cilia formation. Genes Cells **13:** 13-25.

Kunitomo, H., H. Uesugi, Y. Kohara and Y. Lino, 2005 Identification of ciliated sensory neuron-expressed genes in *Caenorhabdits elegans* using targeted pull-down of poly(A) tails. Genome Biol. **6:** R17.

Rex, E., V. Hapiak, R. Hobson, K. Smith, H. Xiao *et al.*, 2005 TYRA-2 (F01E11.5): a *Caenorhabditis elegans* tyramine receptor expressed in the MC and NSM pharyngeal neurons. Journal of Neurochemistry **94:** 181-191.

Rogers, C., V. Reale, K. Kim, H. Chatwin, C. Li *et al.*, 2003 Inhibition of *Caenorhabditis elegans* social feeding by FMRFamide-relate peptide activation of NPR-1. Nature Neuroscience **6:** 1178-1185.

Sagasti, A., O. Hobert, E. R. Troemel, G. Ruvkun and C. I. Bargmann, 1999 Alternative olfactory neuron fates are specified by the LIM homeobox gene *lim-4*. Genes & Dev. **13:** 1794-1806.

Troemel, E. R., J. H. Chou, N. D. Dwyer, H. A. Colbert and C. I. Bargmann, 1995 Divergent seven transmembrane receptors are candidate chemosensory receptors in *C. elegans*. Cell **83:** 207-218.

Xiao, H., V. M. Hapiak, K. A. Smith, L. Lin, R. J. Hobson *et al.*, 2006 SER-1, a *Caenorhabditis elegans* 5-HT2-like receptor, and a multi-PDZ domain containing protein (MPZ-1) interact in vulval muscle to facilitate serotonin-stimulated egg-laying. Developmental Biology **298:** 379-391.
